# Effect of Two Different Drug-Resistant *Staphylococcus aureus* Strains on the Physiological Properties of MAC-T Cells and Their Transcriptome Analysis

**DOI:** 10.3389/fvets.2022.818928

**Published:** 2022-06-24

**Authors:** Lijiao Yan, Yuze Yang, Xiaojun Ma, Lianhua Wei, Xuerui Wan, Zhao Zhang, Jucai Ding, Jie Peng, Guo Liu, Huitian Gou, Chuan Wang, Xiaoli Zhang

**Affiliations:** ^1^College of Veterinary Medicine, Gansu Agricultural University, Lanzhou, China; ^2^Beijing General Station of Animal Husbandry, Beijing, China; ^3^Gansu Provincial Hospital, Lanzhou, China

**Keywords:** MRSA, MSSA, MAC-T cells, adhesion, invasion, apoptosis, transcriptome

## Abstract

*Staphylococcus aureus* (*S. aureus*) is one of the main pathogens causing mastitis in dairy cows. The current work mainly focuses on the pathway of apoptosis induction in MAC-T cells caused by *S. aureus* infection or other factors. However, the physiological characteristics of *S. aureus* infected MAC-T cells and the resulting mRNA expression profile remain unknown particularly in the case of diverse drug resistant strains. Methicillin-resistant *S. aureus* (MRSA) and methicillin-susceptible *S. aureus* (MSSA) strains were used to infect MAC-T cells to investigate this issue. The adhesion, invasion and apoptosis ability of MRSA-infected group and MSSA-infected group was assessed over time (2, 4, 6, 8, and 12 h). After 8 h, the RNA sequencing was conducted on the MRSA-infected and the MSSA-infected with uninfected MAC-T cells as controls. The results showed that the adhesion and invasion ability of MRSA-infected and MSSA-infected to MAC-T cells increased and then decreased with infection time, peaking at 8 h. The adhesion and invasion rates of the MSSA-infected were substantially lower than those of the MRSA-infected, and the invasion rate of the MSSA-infected group was nearly non-existent. Then the apoptosis rate of MAC-T cells increased as the infection time increased. The transcriptome analysis revealed 549 differentially expressed mRNAs and 390 differentially expressed mRNAs in MRSA-infected and MSSA-infected MAC-T cells, respectively, compared to the uninfected MAC-T cells. According to GO analysis, these differentially expressed genes were involved in immune response, inflammation, apoptosis, and other processes. The Kyoto Encyclopedia of Genes and Genomes (KEGG) analysis indicated the following pathways were linked to adhesion, invasion inflammation and apoptosis, including AMPK, FOXO, HIF-1, IL-17, JAK-STAT, MAPK, mTOR, NF-κB, p53, PI3K-Akt, TNF, Toll-like receptor, Rap1, RAS, prion disease, the bacterial invasion of epithelial cells pathway. We found 86 DEGs from 41 KEGG-enriched pathways associated with adhesion, invasion, apoptosis, and inflammation, all of which were implicated in MAC-T cells resistance to MRSA and MSSA infection. This study offers helpful data toward understanding the effect of different drug-resistant *S. aureus* on dairy cow mammary epithelial cells and aid in the prevention of mastitis in the dairy industry.

## Introduction

Mastitis is one of the most common diseases in dairy cows, leading to reduced lactation and, in severe cases, septicemia, gangrene, and the atrophy of udder, sometimes resulting in the permanent loss of lactation capacity and serious economic losses (1). *Staphylococcus aureus* is one of the main pathogens causing mastitis in dairy cows (2). Mastitis induced by *S. aureus* infection in dairy cows was characterized by immunosuppression, subclinical signs, and a long incubation period compared to other bacterial infections (3). In recent years, the misuse of antibiotics has led to the emergence of a large number of drug-resistant bacteria, and 70–90% of bacteria detected in the mastitis caused by *S. aureus* infections exhibit resistance (4, 5). The methicillin-resistant *S. aureus* (MRSA) is the most common drug-resistant pathogen, and its propensity to develop resistance to multiple antibiotics promotes *S. aureus* infections (6). Although much of the attention has been focused on the methicillin-resistant “variant” MRSA, the methicillin-susceptible counterpart (MSSA) is still a major pathogen (7). *Staphylococcus aureus* isolated from the bovine mastitis in Ningxia Hui Autonomous Region of western China was characterized as multidrug-resistant (8). The detection rate of MRSA was 14.7% in *S. aureus* infection with subclinical dairy mastitis in China (9, 10). Biofilm formation was considered to be a major contributor to antibiotic resistance, and interestingly, both MRSA and MSSA have the ability to form biofilms through polysaccharide intracellular adhesion and adhesive matrix molecules (11, 12). However, the adhesion and invasion in mammary epithelial cells by *S. aureus* has been shown to be a key element in the pathogenesis of mastitis (13). The ability of *S. aureus* isolated in bovine mastitis to adhere to and invade bovine mammary epithelial cells has been evaluated (14–17). Also, it was found that most intracellular *S. aureus* were found in MAC-T cell membrane-bound vacuoles, and perhaps this was one of the reasons for their escape from antimicrobial drugs (18). Some MRSA enter the bovine udder epithelium and evade the immune system, causing persistent udder inflammation in cows (19). Moreover, these inflammatory mammary glands exhibit a range of inflammatory features such as tissue damage, cell death, cytokine production, and altered cell proliferation and migration (20, 21).

After the cells were infected by *S. aureus*, they recognize *S. aureus* through plasma membrane Toll-like receptor (TLR2) and subsequently activate signaling pathways such as PI3K-Akt pathway and mTOR pathway to mediate various cellular responses such as apoptosis (22). In bovine mastitis tissue, TLR-mediated pathways cause a series of immune responses (13). In addition, the activated NF-κB was involved in regulating cell proliferation and apoptosis processes and in the production of inflammatory cytokines (23). More importantly, *S. aureus* can activate *Caspase 8* and *Caspase 3* to induce MAC-T cell apoptosis (24, 25). The transcriptome of *S. aureus*-infected MAC-T cells in the exosome has been found to be involved in the signaling pathways associated with bacterial infection (26). The pathogen-specific differential regulation of miRNAs in *S. aureus*-infected MAC-T cells has been previously demonstrated (27). However, the physiological characteristics and mRNA expression of MAC-T cells infected with diverse drug-resistant *S. aureus* are still unknown. Therefore, in this study, we compared the ability of MRSA and MSSA to adhere to and invade MAC-T cells and to induce apoptosis. Meanwhile, based on the multiple pathways of the adhesion invasion, apoptosis, and mastitis caused by *S. aureus* infection, we compared the changes in the expression of MAC-T cell-associated signaling pathways and in differential genes between the MRSA-infected group and the MSSA-infected group by transcriptome analysis. This study provides new data on the mechanism of MAC-T cell infection by different drug-resistant *S. aureus*.

## Materials and Methods

### Bacterial Strains and Growth Conditions

Both MRSA and MSSA strains were provided from Lanzhou Institute of Animal Husbandry and Pharmaceutical Sciences, Chinese Academy of Agricultural Sciences and grown at 37°C in 5 ml of Tryptone Soy Broth (TSB) medium (Beijing, Solarbio) as literature described (9, 10). When its OD_600_ reached to 1.1, MRSA and MSSA strains were collected by centrifugation at 8,000 r/min for 5 min and re-suspended in Dulbecco's Modified Eagle Medium (DMEM) (Hyclone and Sigma) medium without fetal bovine serum (FBS, BI, Israel), bacterial suspension diluted to 10^7^ CFU/ml with DMEM for infection of MAC-T cells.

### Bovine Mammary Epithelial Cells Culture

The MAC-T cells were cultured in warm growth medium consisting of DMEM (Hyclone and Sigma). It was supplemented with 10% heat-inactivated FBS (BI, Israel). The cells were seeded into 25 cm^2^ tissue culture flasks (Corning, China) and incubated at 37°C in a humidified incubator containing 5% CO_2_ until fusion monolayer. The fused monolayers of MAC-T cells were digested with 0.25% trypsin (Gibco, BRL) and inoculated onto 12-well cell culture plates. After 24 h of incubation at 37°C with 5% CO_2_, the cells were starved of DMEM without FBS for 2 h. The cultured cells were used for subsequent experiments.

### Adherence and Invasion Assays in MAC-T cells

For the adhesion and the invasion assays, MRSA and MSSA strains at 10^7^ CFU/ml were co-cultured with confluent monolayer of MAC-T cells (10^5^ cells/ml) in DMEM without FBS at a multiplicity of infection (MOI, ratio of *S. aureus* organisms to cells) of 100:1 at 37°C in 5% CO_2_ for various time periods (2, 4, 6, 8, and 12 h). Then, the supernatants were removed from each well, and each well of cell's monolayers was washed thrice with phosphate buffered saline (PBS) (pH 7.4). For the invasion assay, after *S. aureus*-infected MAC-T cells were washed thrice with 1 ml/well of PBS (pH 7.4), 1 ml of gentamicin-containing medium at a final concentration of 50 mg/ml was added and incubated at 37°C for 2 h to eliminate extracellular bacteria. Then the supernatants were collected and plated on TSB to verify bacterial killing by gentamicin. After the cell–culture medium containing antibiotics were removed, MAC-T monolayers were washed 3 times with PBS. Finally, these treated cells were treated with 0.25% trypsin 0.1% Ethylene Diamine Tetraacetic Acid (EDTA) (Gibco, BRL) and further lysis with Triton X-100 (Amersham, USA). Then MAC-T lysates were serially diluted 10-fold, plated on TSB and incubated overnight at 37°C for detection the number of adherent and invasive bacteria. The colony forming units per milliliter (CFU/ml) of MRSA and MSSA in MAC-T cells were determined by standard colony counting techniques. Each strain and condition were tested in three independent experiments (28).

### The MAC-T Cells Apoptosis Assays

The flow cytometry was performed on MAC-T cells gated on the basis of their forward and side light scatter with any cell debris excluded from analysis. The cells in early apoptosis bind Anexin V but exclude PI (Anexin V+ PI–), while the late apoptotic processes or necrotic cells bind both Anexin V and PI (Anexin V+ PI+), and the necrotic cells were only PI positive (Anexin V– PI+). The apoptosis and necrosis of the uninfected,MRSA-infected and MSSA-infected MAC-T cells were detected by flow cytometry (BD LSRFortessa^TM^ Cell Analyzer, 2010 BD, USA) using the Anneix V-FITC Apoptosis Detection Kit (Bioscience, China). The uninfected,MRSA-infected and MSSA-infected MAC-T cells were incubated for various time periods (2, 4, 6, 8, and 12 h), and then supernatants were removed from each well. After that, the cell monolayers were washed thrice with PBS, and treated with 0.25% trypsin 0.1% EDTA (Gibco) for 3 min. The obtained MAC-T cell suspension was centrifuged at 800 g for 5 min at 4°C, and then re-suspended in 500 μl of 1X annexin-V binding buffer. Then 5 μl of annexin-V FITC and 5 μl of PI were added to the binding buffer, and the cells were incubated for 15 min at room temperature free of light. The MAC-T cells were treated with heat at 55°C for 15 min as a positive control for apoptosis. Finally, these treated MAC-T cells were rapidly detected by flow cytometry (BD LSRFortessa^TM^ Cell Analyzer, 2010 BD, USA). The data were analysis by BD FACSDiva Software (28).

### The RNA Extraction and RNA Sequencing

Total RNA was extracted from uninfected MAC-T cells, MRSA-infected and MSSA-infected MAC-T cells for 8 h using the Trizol (Invitrogen, USA) method. The RNA integrity was precisely detected by 1% agarose gel electrophoresis and Agilent 2,100 bioanalyzer, and RIN numbers ranged from 9.5 to 10. Briefly, mRNA was purified from total RNA by using poly-T oligo-attached magnetic beads. The libraries were constructed by synthesizing cDNA through reverse transcription, end repair and splicing, and PCR amplification steps (29, 30). After the library construction, an initial quantification was first performed using a Qubit 2.0 Fluorometer, and sequencing was performed on Illumina NovaSeq 6,000 platform (Illumina Inc., San Diego, CA) after library inspection was qualified.

### Sequence Data Quality Control and Reads Mapping to the Reference Genome

To ensure the quality and reliability of the data analysis, it is necessary to filter raw data, including the removal of reads with connectors (adapter), the removal of reads containing N (N indicates that the base information cannot be determined) and the removal of low-quality reads (reads with Qphred-value ≤ 20 bases accounting for more than 50% of the entire read length). The quality of the filtered data was assessed using fastp (version 0.19.7). Finally, the index of the reference genome (Bos_taurus_Ensemble_104) was built using Hisat2 (v2.0.5) and paired-end clean reads were aligned to the reference genome using Hisat2 (v2.0.5).

### Differential Expression Analysis

The fragments per kilobase of exon model per million mapped fragments (FPKM) of each gene was calculated using the feature Counts v1.5.0-p3 in the subread software for quantitative analysis. A differential expression analysis of three conditions/groups (three biological replicates per condition) was performed using the DESeq2 R package (1.20.0). The resulting *P*-values were adjusted using the Benjamini and Hochberg's approach for controlling the false discovery rate. The *p*-value < 0.05 and |log_2_ (Fold change)| value > 1 were set as the threshold for significantly differential expression.

### Gene Ontology and Kyoto Encyclopedia of Genes and Genomes Enrichment Analysis of Differentially Expressed Genes

The GO enrichment analysis of DEGs was implemented by the cluster Profiler R package (3.8.1), in which gene length bias was corrected. The cluster Profiler R package (3.8.1) was used to test the statistical enrichment of differential expression genes in KEGG pathways.

### Protein–Protein Interaction Networks Analysis of DEGs and Clustering Analysis of DEGs

The Diamond software (0.9.13) was used to compare the target gene sequence with the selected reference protein sequence, and then the network was established according to the known interaction of the selected reference species. We mapped the interaction network of the screened 86 DEGs using STRING (https://www.string-db.org/) and performed a cluster analysis of these 86 DEGs.

### Real-Time PCR

Based on the National Center for Biotechnology Information (NCBI) gene sequences of β*-actin* (DQ838049.1, *ATP8* (MH576698.1), *CPT1A* (NM_001304989.2), *PLK4* (NM_001083427.2), *IRF7* (BC151518.1), *BOLA-DQB* (Y18201.2), *CTSC* (BC102115.1), *PTGS2* (NM_174445.2), *ITGB3* (NM_001206490.3), the KEGG pathways in which these DEGs were located shown in Table 1. Primers were designed by Prime 5.0 software (Table 2), and the primer sequences were compared by BLAST button in NCBI to verify the primer specificity. β*-actin* was used as an internal reference gene.

**Table 1:** The signaling pathway in which the DEGs were located.

**DEGs**	**Signaling pathways**
PTGS2	IL-17 signaling pathway TNF signaling pathway NF-κB signaling pathway VEGF signaling pathway
CPT1A	PPAR signaling pathway AMPK signaling pathway
ITGB3	PI3K-Akt signaling pathway Rap1 signaling pathway Focal adhesion
PLK4	FOXO signaling pathway
IRF7	NOD-like receptor signaling pathway RIG-I-like receptor signaling pathway Toll-like receptor signaling pathway
ATP8	Oxidative phosphorylation Prion disease
BOLA-DQB	*S. aureus* infection Cell adhesion molecules
CTSC	Apoptosis
ITGB3	Rap1 signaling pathway PI3K-Akt signaling pathway

**Table 2:** The sequence of primer used in this study.

**Name**	**Primer sequence (5-3^**′**^)**	**NCBI no**.
ATP8	Sense: CGTGAACTGACAGTGATCTTATCAA	MH576698.1
	Antisense: GGTTCCGAGAGGGAGACCTA	
CTSC	Sense: TTTGCATTTGCCAACATC	BC102115.1
	Antisense: TCTTGCTTCCATCATCCC	
IRF7	Sense: ACGCCCATCTTTGACTTCG	BC151518.1
	Antisense: CACCAGGACCAGGCTCTTCT	
CPT1A	Sense: CCCCATAATCGTAGGAAG	NM_001304989.2
	Antisense: TTTGGAGAAGCAGCACTA	
BOLA-DQB	Sense: TGACCCTGGTGATGCTGA	Y18201.2
	Antisense: CGTCCCGTTGGTGAAGTA	
PLK4	Sense: CTACGATTCCGCTCTGCT	NM_001083427.2
	Antisense: ATGTGCTTGCTGTCCCTC	
PTGS2	Sense: GCCTGGTCTGATGATGTA	NM_ 174445.2
	Antisense: GATTAGCCTGCTTGTCTG	
ITGB3	Sense: GACATCCTGGTGGTCTTGCT	NM_001206490.3
	Antisense: AAGTGCCCCTGTAGGTGATG	
β-actin	Sense: TCAACGGGAAGCTCACTGG Antisense: CCCCAGCATCGAAGGTAGA	DQ838049.1

The total cellular RNA was extracted from MRSA-infected and MSSA-infected MAC-T cells for 8 h using the Trizol (Vazyme, China) method. cDNA was obtained by reverse transcriptase reagent according to the HiScript II 1st Strand cDNA Synthesis Kit (+gDNA wiper. Vazyme. China) instructions. Reaction system included 4 μl 4X gDNA wiper Mix, 1 μg template RNA, RNase-free ddH_2_O to 16 μl, 42°C for 2 min, followed by the addition of 4 μl 5X HiScript II superMix, 50°C for 5 min, 86°C for 5 s. We performed qRT-PCR using a LightCycler96 (Roche, Switzerland) instrument, and water as a negative control. Each reaction volume was 20 μl, including 10-μl SYBR Premix Ex Taq II (2×), 0.4 μl of upstream primer (10 mM), 0.4 μl of downstream primer (10 mM), and 4.0 μl of cDNA template (300 ng). The followed procedures were as follows: 300 s at 95°C, 10 s at 94°C, 30 s at 57°C, and 35 cycles of 20 s at 72°C. We analyzed the data using LightCycler® 96 software (version 1.1.0.1320), and the relative expression values of the genes were calculated using the 2^−ΔΔCt^ method and the normalization method.

### Western Blot.

After the MAC-T cells were infected by MRSA and MSSA for different time periods, they were washed thrice with ice-cold PBS and the proteins were extracted using high efficiency RIPA cell lysis solution (Solarbio, China). The protein concentration was determined using the BCA protein analysis reagent (Vazyme, China). The equal amount of proteins (30 μg) was separated from each sample using 12% sodium dodecyl sulfate–polyacrylamide gels (SDS–PAGE) and transferred to polyvinylidene fluoride (PVDF) (GE Healthcare, Wasukesha, WI, USA). We performed SDS–PAGE on two gels in the same electrophoresis machine at the same time to ensure the reliability of the results. The membranes were blocked with skimmed milk containing 0.05% Tween-20 (TBS-T) and then incubated with anti-PrP (1:1,500, Abcam, UK), anti-p65 (1:1,000, Proteintech Group, Inc.), anti-tumor necrosis factor α (anti-TNF-α) (1:1,000, Bioss, China), anti-interleukin-6 (IL-6) (1:1,000, Bioss, China), anti-Bcl-2 (1:5,000, Proteintech Group, Inc.), anti-cleavage–Caspase-3 (1:1,500, Cell Signaling Technology, Danvers, USA), anti-P-NF-κB-p65 (1:1,500, Proteintech, USA) and anti-Caspase3 (1:1,000, Proteintech Group, Inc.) primary antibodies overnight at 4°C, with β-actin (1:5,000, Bioss, China) as control. The next day, the membranes were washed thrice with TBST buffer for 30 min. After that, they were incubated with goat anti-rabbit IgG H&L (HRP) (1:5,000, Bioss, China) for 2 h at room temperature, and then exposed using chemiluminescence (Amersham Imager 600, USA). The protein bands thus obtained was analyzed by AI600 software. We further analyzed the grayscale values of the protein bands using Image J (version 1.53f51) software (31).

### Statistical Analysis

All the experiments were performed in triplicate, and the representative data were obtained from three independent experiments. Statistical analysis was performed using GraphPad Prism 8. One-way analysis of variance (ANOVA) was used to detect statistical differences between the control group and the MRSA and MSSA treatment groups.

## Results

### Adherence and Invasion to MAC-T Cells by MRSA and MSSA

**Figure 1:**
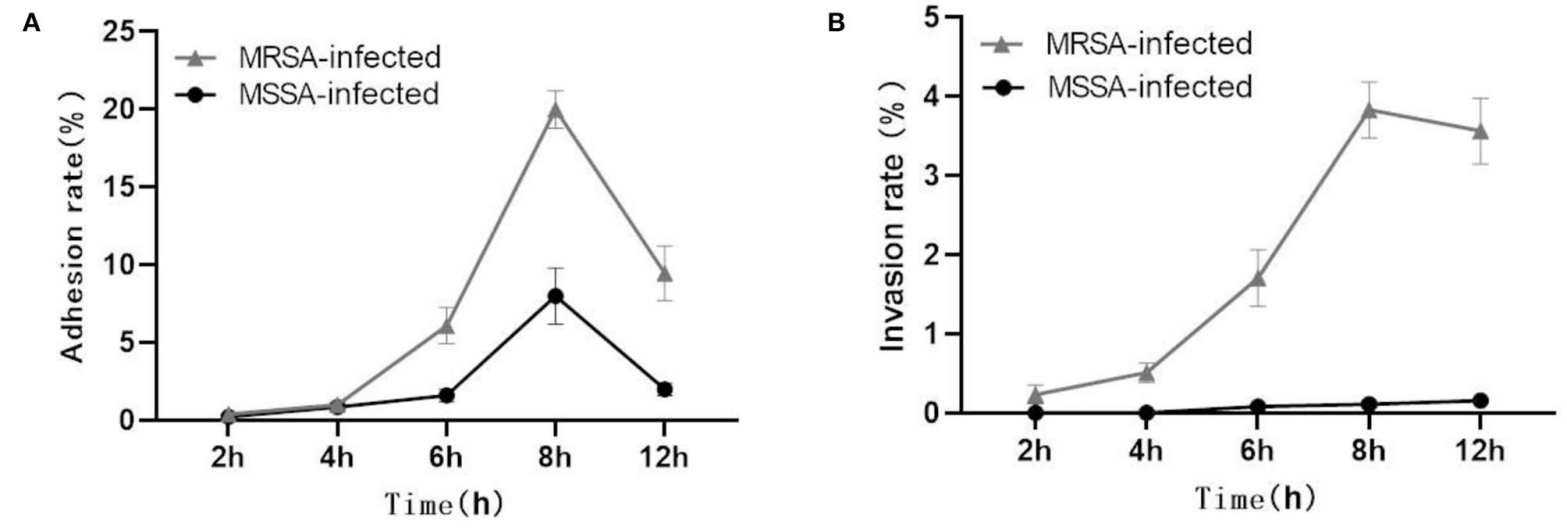
Adhesion and invasion rates in MRSA-infected group and MSSA-infected group. **(A)** The adhesion rate of MRSA-infected and MSSA-infected MAC-T cells. **(B)** The invasion rate of MRSA-infected and MSSA-infected MAC-T cells.

Both MRSA and MSSA were able to adhere to and invade MAC-T cells at different degrees, but MRSA had a stronger ability to adhere to and invade MAC-T cells. The results showed that the adhesion rate of MRSA-infected MAC-T cells increased from 0.5% at 2 h to 20% at 8 h and then decreased to 11.4% at 12 h (Figure 1A). Then the invasion rate increased from 0.3 to 4.0% and then decreased to 3.1%. The adhesion rate of MSSA-infected MAC-T cells increased from 0.3% at 2 h to 9.5% at 8 h and then decreased to 2.4% at 12 h, and importantly, the invasion rate of MSSA-infected MAC-T cells increased from 0.005 to 0.20% with time but did not change significantly (Figure 1B).

### The MRSA and MSSA Induced Apoptosis in MAC-T Cells

**Figure 2:**
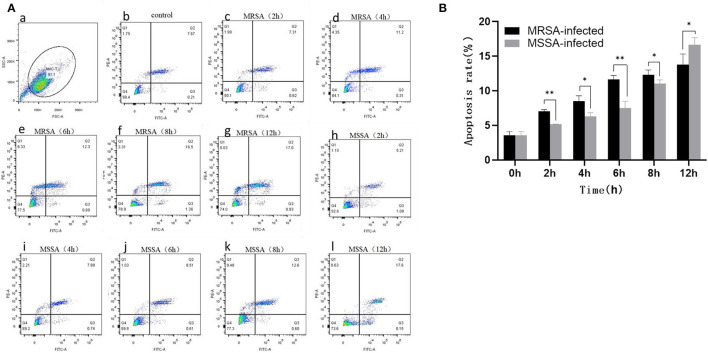
The apoptosis rates of MRSA-infected and MSSA-infected MAC-T cells at different time periods. **(A)** The apoptosis rate of cells measured by flow cytometry. Q1 indicates necrotic cells (Anexin V– PI+), Q2 indicates late apoptotic cells (Anexin V+ PI+), Q3 indicates surviving cells (Anexin V– PI–), and Q4 indicates early apoptotic cells (Anexin V+ PI–). (a) The application of gating strategy in the analysis of MAC-T cell apoptosis. (b) The apoptosis rate of uninfected MAC-T cells. (c)–(g) The apoptosis rates of MRSA-infected cells at different time periods (2, 4, 6, 8, and 12 h). (h)–(l) The apoptosis rates of MRSA-infected cells at different time periods (2, 4, 6, 8, and 12 h). **(B)** The percentage of MRSA and MSSA induced apoptosis in MAC-T cells with time. *p < 0.05, **p < 0.01.

Both MRSA-infected and MSSA-infected MAC-T cells were assayed with fluorescently labeled membrane-linked protein V (Annexin-V) and propidium iodide (PI). As showed in Figure 2, both MRSA and MSSA were able to induce different degrees of apoptosis in MAC-T cells. The apoptosis rate of MAC-T cells tended to increase over time, with that of MRSA-infected cells increasing from 7 to 13% and that of MSSA-infected cells from 5 to 16%. Interestingly, the apoptosis rate of MRSA-induced cells before 8 h was significantly higher than that of the MSSA-infected. However, the apoptosis rate of MSSA-induced group at 12 h was significantly higher than that of the MRSA-infected group.

### Analysis of Total DEGs in Both MRSA-Infected and MSSA-Infected MAC-T Cells

**Figure 3:**
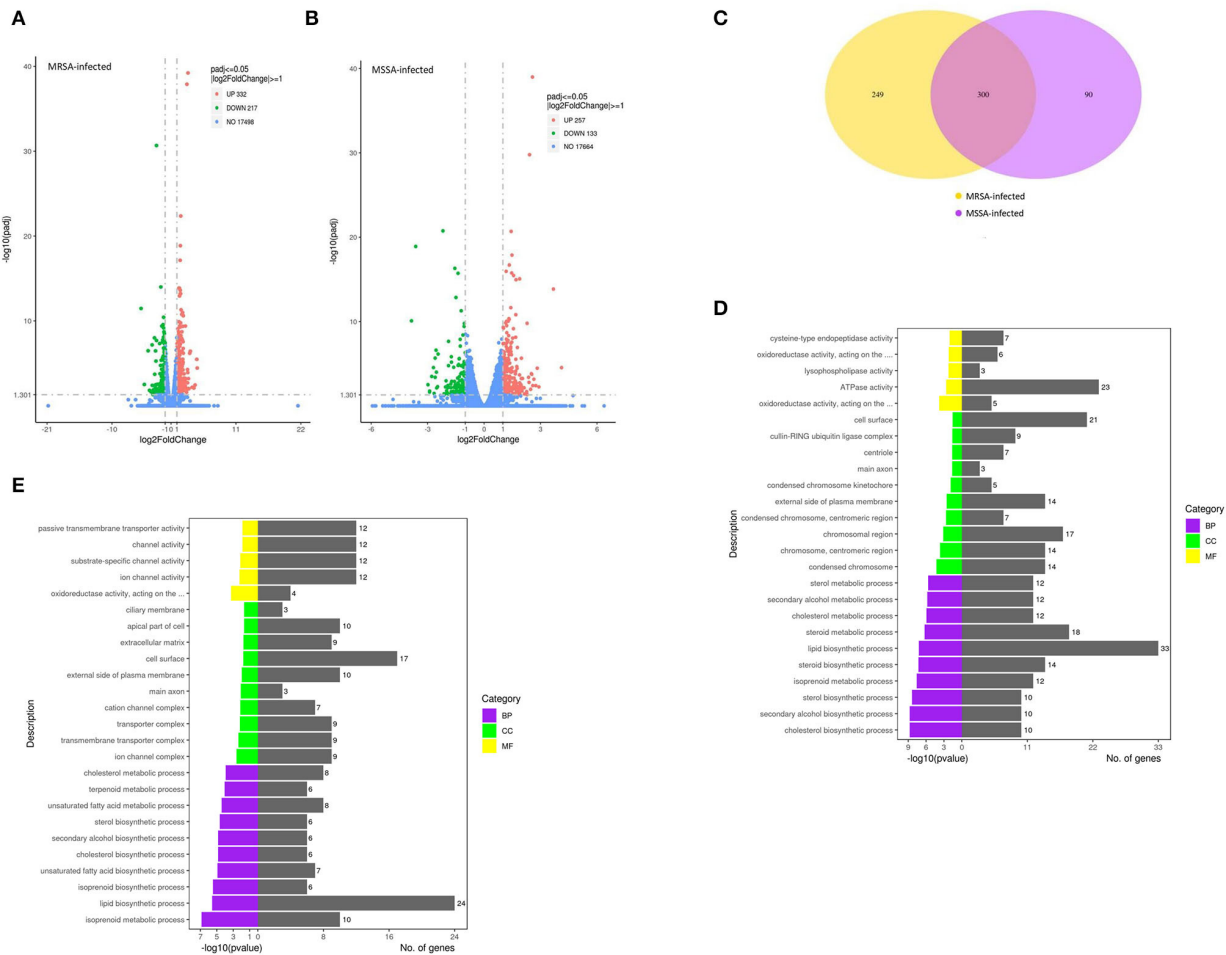
The functional enrichment analysis of total DEGs. **(A)** and **(B)** The volcano plots of total differentially expressed genes of MRSA-infected and MSSA-infected MAC-T cells. Log_2_ |Fold change| >1 and *p* < 0.05 are the thresholds. Red plots represent up-regulated DEGs and green plots represent down-regulated DEGs. **(C)** The Venn diagram of total differentially expressed genes. Purple represents MRSA-infected group. Yellow represents MSSA-infected group. **(D)** and **(E)** The GO functional enrichment pathway of MRSA-infected and MSSA-infected MAC-T cells.

In this study, we selected differentially expressed genes through two levels of multiple of difference (|log_2_ (Fold change) | > 1) and significance level (*p* < 0.05) by DESeq2. There were 549 DEGs in the MRSA-infected group, of which 332 genes were significantly up-regulated and 217 genes were significantly down-regulated (Figure 3A); there were 390 DEGs in the MSSA-infected group, of which 257 genes were significantly up-regulated and 133 genes were significantly down-regulated (Figure 3B). The 549 DEGs of the MRSA-infected group were enriched in 274 signaling pathways, with up-regulated genes enriched in 231 of these pathways and down-regulated genes in 165 of them. The 390 DEGs of the MRSA-infected group were enriched in 241 signaling pathways, with up-regulated genes enriched in 199 of these pathways and down-regulated genes in 144 of them. There were 300 DEGs in both the MRSA-infected and MSSA-infected MAC-T cells (Figure 3C).

### The GO Analysis of Total DEGs

To fully analyze the cellular potential functional changes induced by both MRSA and MSSA infection of cells, we performed GO enrichment analysis of DEGs. As showed in Figures 3D,E, the GO analysis of total DEGs helped further analyze the differences in genes expression between the MRSA-infected and the MSSA-infected MAC-T cells in terms of the following three aspects: Biological processes, cellular components, and molecular functions. With GO functional analysis, these DEGs were mainly involved in cellular processes, developmental processes, cell growth, localization, organization of cellular components, cell death, metabolic processes and biological regulation (Figures 3D,E).

### KEGG Enrichment Analysis of Associated With Adhesion, Invasion Apoptosis, and Inflammation

**Figure 4:**
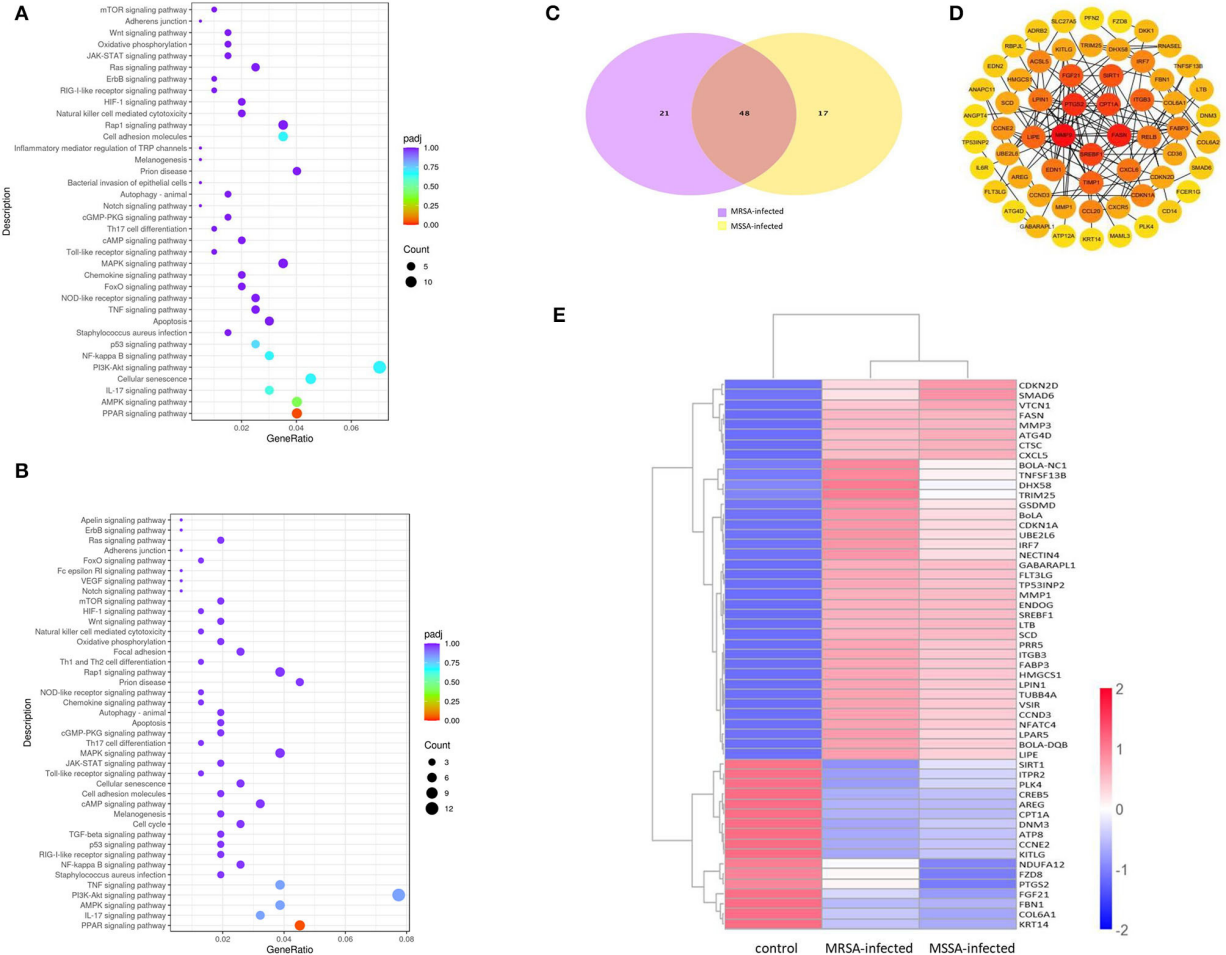
The enrichment analysis of selected DEGs in MRSA and MSSA infection groups. **(A)** and **(B)** The KEGG enrichment analysis of selected DEGs in MRSA and MSSA infection groups. **(C)** The Venn diagram of screening DEGs. Purple indicates MRSA-infected, yellow indicates MSSA-infected. **(D)** The interaction network map of important DEGs. **(E)** The clustering of important differentially expressed genes. Red represents highly expressed genes and blue represents lowly expressed genes.

To explore the KEGG pathway linked to adhesion, invasion, apoptosis, and inflammation, we screened 36 relevant KEGG signaling pathways in the MRSA-infected group, and 41 relevant KEGG signaling pathways in the MSSA-infected group (Figures 4A,B). The bacterial invasion of epithelial cells and inflammatory mediator regulation of TRP channels were present only in the MRSA-infected group; Apelin signaling pathway, cell cycle, Fc epsilon RI signaling pathway, focal adhesion, TGF-β signaling pathway, Th1 and Th2 cell differentiation, and VEGF signaling pathway were present only in the MSSA-infected group. There were 34 KEGG pathways in both the MRSA-infected and MSSA-infected MAC-T cells, including apoptosis, the bacterial invasion of epithelial cells, prion disease, oxidative phosphorylation, cell adhesion molecules, focal adhesion, AMPK, FOXO, JAK-STAT, MAPK, PI3K-Akt, IL-17, NF-κB, Rap1, RAS, TNF, HIF-1, Toll-like receptor and Chemokine signaling pathway (Figures 4A,B). At the same time, we found that MRSA and MSSA infection of MAC-T cells could activate the prion disease pathway. A total of 86 DEGs were screened from these KEGG pathways, and the signal pathways of MRSA-infected and MSSA-infected MAC-T cells were different. More importantly, the genes expressed were different in the same signal pathway (Table 3).

**Table 3:** KEGG pathway enrichment analysis of MRSA-infected and MSSA-infected MAC-T cells.

**KEGG pathway**	**Common gene**	**Specific gene**	
		**MRSA specific genes**	**MSSA specific genes**
HIF-1signaling pathway	IL6R	CDKN1A/ANGPT4/ALDOC	EDN1
RAS signaling pathway	FLT3LG/KITLG/FGF21	ANGPT4	
Apoptosis	CTSC/ENDOG/BBC3/	ITPR2/PRF1	
Bacterial invasion of epithelial cells		DNM3	
Chemokine signaling pathway	CXCL5/CXCR5	CCL20	
Prion disease	ATP8/TUBB4A/CREB5	ITPR2	NDUFA12/UBE2L6/FZD8/PTGS2
Cellular senescence	CCND3/BOLA/CCNE2/NFATC4/	SIRT1/ITPR2/CDKN1A/BOLA-NC1	
AMPK signaling pathway	SCD/CPT1A/SIRT1/LIPE/CREB5/CD36	FASN/SREBF1	
FOXO signaling pathway	GABARAPL1	SIRT1/PLK4/CDKN1A	CDKN2D
JAK-STAT signaling pathway	CCND3/IL6R	CDKN1A	
MAPK signaling pathway	AREG/FLT3LG/KITLG/CD14/FGF21	ANGPT4/RELB	CACNA1E
PI3K-Akt signaling pathway	CCND3/AREG/ITGB3/CCNE2/LPAR5/CREB5	CDKN1A/ANGPT4	
PI3K-Akt signaling pathway	FLT3LG/KITLG/COL6A1/FGF21/IL6R/COL6A2		
cAMP signaling pathway	CREB5/LIPE/EDN2		ADRB2/EDN1
IL-17 signaling pathway	MMP3/MMP1/MMP9	CXCL5/CCL20	PTGS2/CXCL5
NF-κB signaling pathway	LTB/CD14/CARD14	TRIM25/TNFSF13B/RELB	PTGS2
NOD-like receptor signaling pathway	GABARAPL1/IRF7	GSDMD/ITPR2/RNASEL	
Toll-like receptor signaling pathway	IRF7/CD14		
Oxidative phosphorylation	ATP8/ATP12A		NDUFA12
Adherens junction	NECTIN4		
Cell adhesion molecules	BOLA/BOLA-DQB/VTCN1	VSIR/BOLA-NC1	
Focal adhesion			CCND3/ITGB3/COL6A1/COL6A2
Rap1 signaling pathway	ITGB3/LPAR5/KITLG/FGF21/PFN2	ANGPT4	SKAP1
TNF signaling pathway	MMP3/CREB5/CXCL5/MPP9	CCL20	PTGS2/EDN1

### Enrichment Analyses of DEGs Associated With Adhesion, Invasion, and Apoptosis

We selected 86 DEGs in the MRSA-infected and MSSA-infected MAC-T cells, there were 68 DEGs in MRSA-infected group, of which 48 genes were significantly up-regulated and 20 genes were significantly down-regulated. 64 DEGs were in MSSA-infected group, of which 41 genes were significantly up-regulated and 23 genes were significantly down-regulated (Figure 4C). To compare the expression differences of the same genes in the MRSA-infected and MSSA-infected groups, we used the FPKM of differential genes as the expression levels and used different colored regions to indicate different clustering grouping information. The hierarchical clustering analysis of DEGs is shown in Figure 4E. Then, we made interaction network plots of the 86 DEGs and found a total of 60 reciprocal nodes, and the genes with the strongest interactions were *PTGS2, CPT1A, FANS, SREBF1*, and *MMP9* (Figure 4D).

### Results of RT-qPCR Validation Was in Accordance With RNA-seq

**Figure 5:**
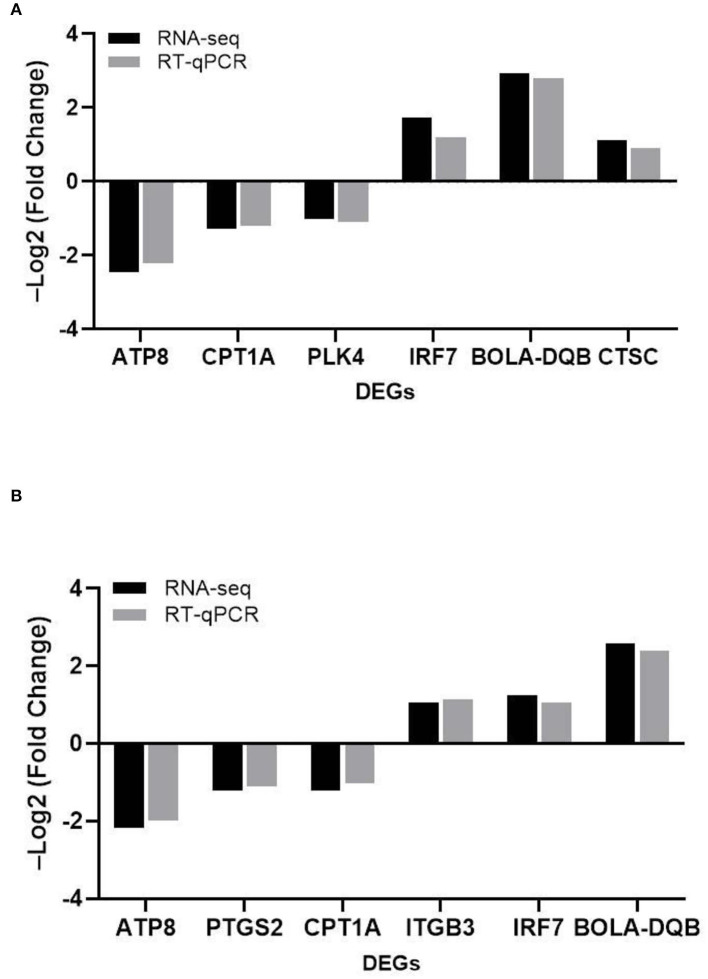
The comparison of the relative expression of DEGs screened by RT-qPCR and RNA-seq for the MRSA-infected and MSSA-infected groups. **(A)** RT-qPCR to detect the relative expression of DEGs screened from the MRSA-infected group. **(B)** RT-qPCR to detect the relative expression of DEGs screened from the MSSA-infected group.

To verify the reliability of the transcriptome results, we randomly selected DEGs associated with adhesion, invasion, apoptosis, prion disease, and inflammation for validation RT-PCR (Table 1). The differentially expressed genes contained in the MRSA-infected group included *ATP8, CPT1A, PLK4, IRF7, BOLA-DQB, CTSC*, and those in the MSSA-infected group included *ATP8, PTGS2, CPT1A, ITGB3, IRF7, BOLA-DQB*. The RT-qPCR results were consistent with the RNA-seq results (Figures 5A,B), indicating that the RNA-seq results were reliable.

### The MRSA and MSSA Activate the Inflammatory Response of MAC-T Cells and Induce Apoptosis

**Figure 6:**
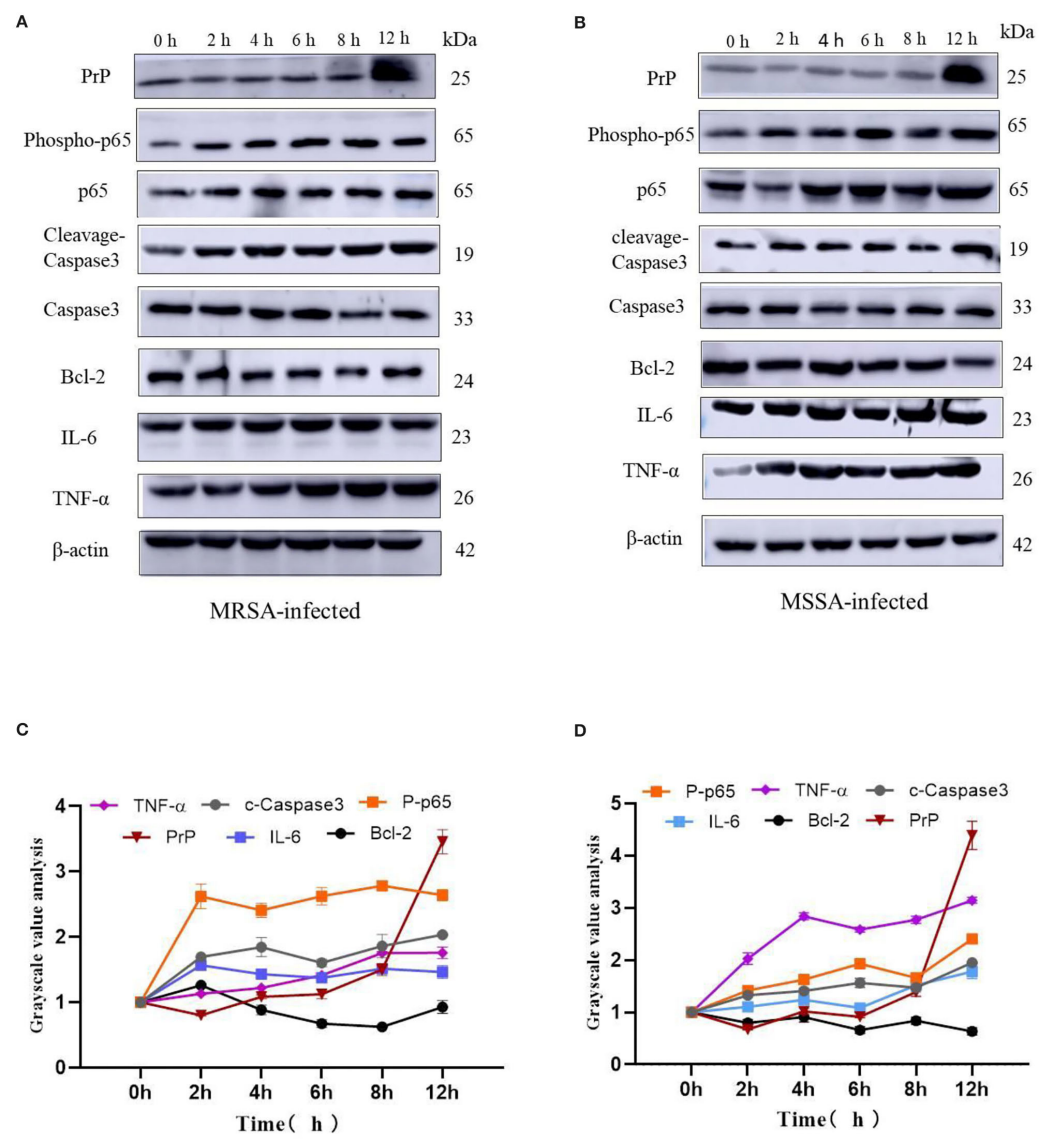
The effects of MRSA-infected and MSSA-infected MAC-T cells on the expression levels of TNF-α, IL-6, Bcl-2, cleavage–Caspase3, Phospho-p65 and PrP. **(A)** and **(B)** The MRSA-infected group and MSSA-infected group on the expression levels of TNF-α, IL-6, Bcl-2, cleavage–Caspase3, Phospho-p65, and PrP by Western blotting. **(C)** and **(D)** Gray-scale analysis of MRSA-infected group and MSSA-infected group.

The MRSA and MSSA infection of MAC-T cells could activate prion disease, apoptosis, and inflammation-related signaling pathways. Therefore, we examined the expression levels of TNF-α, IL-6, Bcl-2, cleaved-Caspase3, Phospho-p65, and PrP by Western blotting. The results indicated that MRSA and MSSA treatment of MAC-T cells increased the protein expression of TNF-α, IL-6, cleavage–Caspase3, Phospho-p65, and PrP, and then the protein expression of Bcl-2 appeared to decrease slightly with the duration of infection (Figures 6A,B). In the MRSA-infected group, the phosphorylated p65 and IL-6 expression peaked at 2 h after infection, and there was no significant difference in expression from 4 to 12 h compared to 2 h. With the increasing MRSA-infected time, the protein expression of cleavage–Caspase3 and TNF-α rose progressively and peaked at 12 h. The level of Bcl-2 protein decreased as infection duration increased, with the lowest expression at 8 h. Then the expression of PrP increased throughout time, reaching a peak of 3.5 times higher than that of the uninfected at 12 h (Figures 6A,C). In the MSSA-infected group, the protein expression of phosphorylated p65 increased gradually with increasing infection time, peaking at 12 h. The TNF-α expression tended to rise with infection time, but showed a minor reduction at 6 h after infection and subsequently surged again, peaking at 12 h. The cleavge-Caspase3 and IL-6 protein expression rose as infection time increased, peaking at 12 h. With the increasing infection duration, Bcl-2 expression declined steadily, with the lowest expression at 12 h. The PrP expression was also 4.6 times higher in the experimental group than in the control group (Figures 6B,D). Importantly, the MSSA-infected group was higher in the expression of TNF-α, IL-6, and Bcl-2 compared to the MRSA group. All these confirm that there were differences in the mechanisms by which MRSA and MSSA activate cellular immune responses and induce apoptosis.

## Discussion

*Staphylococcus aureus* can cause chronic mastitis in dairy cows (2). *Staphylococcus aureus* infection can cause mammary gland damage, impair antimicrobial immune response, promote bacterial transmission, and it is frequently linked with mammary gland death and the induction of apoptosis (32). One of the major reasons for persistent infection was the development of bacterial resistance and the capacity to avoid identification by the host immune system during the *S. aureus*-infected (19). The recent researches have showed that the bacteria's capacity to adhere to cells was directly related to their pathogenicity. Importantly, their adhesion to mammary epithelial cells was a critical stage in invading host cells (13, 33, 34) and the invasion may protect the bacteria from being eliminated by the immune system and persist in chronically infected hosts (35). Furthermore, the pathogen-targeted differential regulation of miRNAs in MAC-T cells has been identified by transcriptome. The regulation played a role in immunity and development, which verified the involvement of mammary epithelial cells in the immune response to invading pathogens (27). The differentially expressed genes, mRNAs and lncRNAs in the transcriptional profile of exosomes were discovered to be engaged in the signaling pathways associated with bacterial invasion and adhesion, oxidative stress, inflammation and apoptosis (26). To investigate how MAC-T cells resisted the infection by different drug-resistant *S. aureus* strains, we assessed the similarities and differences in the ability to adhere to, invade and induce apoptosis in MAC-T cells by MRSA and MSSA using transcriptome.

This study found that both MRSA and MSSA could adhere to and invade MAC-T cells at different degrees, with MRSA had a stronger ability to adhere to and invade MAC-T cells. It has been demonstrated that various strains have different abilities to adhere to and invade cells (28). In this study, the adhesion and invasion rates of MRSA-infected MAC-T cells gradually increased with time, peaking at 8 h and then declining. The decline was most probably caused by the prolonged bacterial action on cells, which led to cell and bacterial apoptosis. As a result, both adhesion rate and invasion rate decreased. This was in general consistent with the results of other studies where the number of bacteria surviving inside the cells decreased over time (28). Furthermore, the adhesion and the invasion rate of MSSA-infected MAC-T cells were significantly lower than that of MRSA-infected MAC-T, and the invasion rate of MSSA-infected MAC-T cells did not vary significantly over time. KEGG analysis indicated that the MSSA-infected group lacked an enrichment pathway for bacterial invasion of epithelial cells. However, in the MRSA-infected group, this pathway was present, and the *DNM3* expression gene was up-regulated. Although the bacterial invasion of epithelial cells pathway was also present in the studies on exosomal transcriptional profiling, *CD2AP* of DEGs was different from the gene in present study (26). These results suggested that *CD2AP* and *DNM3* genes were associated with the resistance to different *S. aureus* infection. The results are consistent with the persistence of small colony variants of *S. aureus* in bovine mammary epithelial cells (36). The signaling pathways linked to MRSA-infected group and MSSA-infected group adhesion included adherens junction (*NECTIN4*), cell adhesion molecules (*VSIR, BOLA, BOLA-NC1, VTCN1*), Focal adhesion (*CCND3, ITGB3, COL6A, COL6A2*), Rap1 signaling pathway (*ITGB3, LPAR5, KITLG, FGF21, PFN2, ANGPT4, SKAP1*), among which *VSIR, BOLA-NC1*, and *ANGPT4* were unique DEGs in MRSA-infected group, and *CCND3, ITGB3, COL6A1, COL6A2*, and *SKAP1* were unique DEGs in MSSA-infected group. The cell adhesion pathway in *S. aureus* infection was similar to that in other studies, but the DEGs were different (27). The difference in these specific DEGS might be one of the important reasons for the different adhesion rates of MRSA-infected group and MSSA-infected group.

The ability of MRSA and MSSA to induce apoptosis in MAC-T cells was detected by flow cytometry. The results revealed a significant difference in the induction of apoptosis by MRSA and MSSA. Interestingly, the ability of MRSA to induce apoptosis was stronger than that of MSSA before 8 h, while the ability of MSSA to induce apoptosis at 12 h was stronger than that of MRSA. This result suggested that MRSA invasion and colonization in cells might be one of the reasons for the decrease of MRSA activity and the decrease of apoptosis rate, and other authors have also supported this conclusion (28). Under certain conditions, the infection with different drug-resistant *S. aureus* induced the apoptosis of MAC-T cells in a time-dependent manner, and this was consistent with the study on the early apoptosis of primary mammary epithelial cells induced by *S. aureus* infection (25). Furthermore, different bacterial activity may induce cell apoptosis at different degrees (37), which demonstrates that the induction of MAC-T cells apoptosis in by MRSA and MSSA were also different. *S. aureus*, the major pathogenic microorganism, derived from lipoteichoic acid (LTA) has been identified to activate inflammatory responses, and LTA enhanced the messenger RNA (mRNA) expression, the production of TNF-α and IL-6 (38). In this study, MAC-T cells were treated with MRSA and MSSA at different time points: The expression of TNF-α, IL-6, phosphorylated p65, and cleaved-Caspase3 expression varies with the time of infection. Importantly, the protein expression of phosphorylation level of p65 was higher in the MRSA-infected group compared to the MSSA group. In addition, the expression of TNF-α at 12 h was higher in the MSSA-infected group than in the MRSA-infected group. These results indicated that the inflammatory response and apoptosis of MAC-T cells activated by MRSA and MSSA were different. We screened 36 KEGG signaling pathways associated with apoptosis and inflammation by the transcriptome, these important pathways included Toll-like receptor, PI3K-Akt, NF-κB, NOD-like receptor, RAS, MAPK, AMPK, FOXO, JAK-STAT, and apoptosis pathway. Toll-like receptor pathway plays an important role in immune regulation and cellular recognition of bacterial infections (39). After *S. aureus* infection of cells, cells recognize *S. aureus* via the plasma membrane TLR2. This results in increased levels of, cytokines, soluble *CD14* and lipopolysaccharide-binding protein (LBP) during recognition, and in the activation of PI3K/Akt-mTOR signaling pathways, which mediate various cellular responses such as inflammation and apoptosis (21). Similarly, the *CD14* expression in the Toll-like receptor pathway was also up-regulated in this study, and *CD14* can help specific TLRs bind ligands, triggering multiple kinase recruitment signals that eventually participate in the NF-kB, PI3K-Akt, and JAK-STAT signaling pathways (40). The PI3K-Akt pathway was intimately related to cell metabolism, survival and reproduction (41). The LTA of *S. aureus* can trigger the inflammatory response via the PI3K-AKT signaling pathway, whereas miR-23a inhibits the inflammatory response by targeting PI3K (38). In the PI3K-AKT pathway of the MRSA-infected group, *CDKN1A* expression was increased whereas *ANGPT4* expression was down-regulated in this study. In contrast, *CDKN1A* and *ANGPT4* genes were absent in the MSSA-infected group. However, the relevance of these two genes in cellular resistance to bacterial infection was controversial. Among the several inflammation-related signaling pathways, the NF-κB pathway was regarded as particularly significant; activated NF-κB was involved in the regulation of cell proliferation and apoptosis, and in the generation of inflammatory cytokines (42, 43). Furthermore, recent research has revealed that activated NF-κB pathway could trigger as an upstream activator of the NOD-like receptor pathway (43, 44). Then *CTSC, ENDOG, BBC3, ITPR2*, and *PRF1* were among the apoptosis pathway found to be differentially changed in this study. Also, *ITPR2* and *PRF1* in apoptosis pathway were only found in the MRSA-infected group and were not found in the MSSA-infected group. The different rates of apoptosis induction by MRSA and MSSA could be attributed to these discrepancies in DEGs. In fact, the bacterial induction of apoptosis does not activate a single signaling pathway on its own. On the contrary, each signaling pathway interacts with the others to help the cell resist bacterial infection. Finally, *S. aureus* induces MAC-T cell apoptosis via *caspase8* associated with the FAS–FADD death receptor and the apoptosis executor *Caspases3* (24, 25). More importantly, it was also worth noting that MRSA and MSSA infection of MAC-T cells was capable of activating the prion disease pathway. The misfolding of cellular prion protein (PrP^C^) into scrapie isoform of prion protein (PrP^Sc^) in organisms, and it was accumulated in neural cells, which led to the development of prion disease (45, 46). The PrP^C^ was encoded the prion protein gene (*PRNP*) that was a highly conserved and widely expressed cell surface glycoprotein in all mammals (47, 48). The PrP^C^ has been found to function in a variety of cellular pathways and signaling processes throughout the body in humans and animals (49). The *PRNP* was involved in cell adhesion and anti-apoptosis mechanisms (50, 51). Furthermore, PrP^C^ also played a role in the process of bacterial infection. *Helicobacter pylori* infection led to the upregulation of gastric PrP^C^ expression (52). The PrP-knockout mice showed a considerable decrease in a *streptococcal* sepsis with respect to colony counts from the spleen and peripheral blood (53). Also, *Brucella abortus* was capable of interacting directly or indirectly with cellular prion protein on macrophages (54). *Mycobacterium bovis* infection led to a gradual increase in *PRNP* mRNA level, and *PRNP* disruption significantly increased the rate of apoptosis in BV2 microglia infected with *M. bovis* (55). In this study, MAC-T cells were treated with MRSA and MSSA at different time points, and the expression of PrP was significantly increased at 12 h. These results indicated that the *PRNP* might be involved in cellular resistance to bacterial infection. In summary, the physiological properties of adhesion and invasion and apoptosis of MAC-T cells were considerably different by different drug-resistant *S. aureus* infection.

## Conclusions

The MRSA had a stronger ability to adhere to and invade MAC-T cells than MSSA, especially the invasion ability. The ability of MRSA to induce apoptosis before 8 h was stronger than that of MSSA, whereas the ability of MSSA to induce apoptosis after 8 h was stronger than that of MRSA. We analyzed 41 KEGG pathways associated with adhesion, invasion, apoptosis, and inflammation by transcriptome and got 86 DEGs from them in MAC-T cells resistance to different drug-resistant *S. aureus* infections. The data obtained in this study would be useful in understanding the mechanisms by which MAC-T cells resist different drug-resistant *S. aureus* infections.

## Data Availability Statement

The datasets presented in this study can be found in online repositories. The names of the repository/repositories and accession number(s) can be found below: https://www.ncbi.nlm.nih.gov/bioproject/PRJNA778892/.

## Author Contributions

LY, XZ, and CW were involved in the conception and design of this study. LY, XM, JP, GL, XW, ZZ and JD performed the pilot project. LY wrote the first draft of the manuscript. All authors were involved in revising, reading, and approving the submitted version of the manuscript.

## Funding

This study was supported by the Subject Construction Funding of Gansu Agricultural University, Gansu Province, China (GSAU-XKJS-2018-074), Key Research and Development Project Plan of Gansu Provincial Science and Technology Department (18YF1NA077), and the National Natural Science Foundation of China (No. 32060822 and 81960386).

## Conflict of Interest

The authors declare that the research was conducted in the absence of any commercial or financial relationships that could be construed as a potential conflict of interest.

## Publisher's Note

All claims expressed in this article are solely those of the authors and do not necessarily represent those of their affiliated organizations, or those of the publisher, the editors and the reviewers. Any product that may be evaluated in this article, or claim that may be made by its manufacturer, is not guaranteed or endorsed by the publisher.

## Abbreviations

MAC-T: bovine mammary epithelial cell line
DEGs: differentially expressed genes
FOXO: fork head box O
GO: gene ontology
IL: interstitial
KEGG: Kyoto Encyclopedia of Genes and Genomes
MAPK: mitochondria-activated protein kinase
NF-κB: nuclear transcription factor kappa B
PI3K: phosphoinositide 3-base enzyme.

## References

[1] Szyda J Mielczarek M Fraszczak M Minozzi G Williams JL. Wojdak–Maksymiec K. The genetic background of clinical mastitis in Holstein-Friesian cattle. Animal. (2019) 13:2156–63. 10.1017/S1751731119000338 30835192

[2] Zecconi A Scali F. *Staphylococcus aureus* virulence factors in evasion from innate immune defenses in human and animal diseases. Immunol Lett. (2013) 150:12–22. 10.1016/j.imlet.2013.01.004 23376548

[3] Günther J Petzl W Bauer I Ponsuksili S Zerbe H Schuberth HJ. Differentiating *Staphylococcus aureus* from Escherichia coli mastitis: *S. aureus* triggers unbalanced immune-dampening and host cell invasion immediately after udder infection. Sci Rep. (2017) 7:4811. 10.1038/s41598-017-05107-4 28684793 PMC5500526

[4] Wall RJ Powell AM Paape MJ Kerr DE Bannerman DD Pursel VG. Genetically enhanced cows resist intramammary *S. aureus* infection. Nat Biotechnol. (2005) 23:445–51. 10.1038/nbt1078 15806099

[5] Ruegg PL. A 100–year review: mastitis detection, management, andprevention. J Dairy Sci. (2017) 100:10381–97. 10.3168/jds.2017-13023 29153171

[6] Han S Li X Liu J Zou Z Luo L Wu R. Bta-miR-223 Targeting CBLB contributes to resistance to *Staphylococcus aureus* mastitis through the PI3K/AKT/NF-κB pathway. Front Vet Sci. (2020) 7:529. 10.3389/fvets.2020.00529 33195489 PMC7475710

[7] Monaco M Pimentel de Araujo F Cruciani M Coccia EM Pantosti A. Worldwide epidemiology and antibiotic resistance of *Staphylococcus aureus*. Curr Top Microbiol Immunol. (2017) 409:21–56. 10.1007/82_2016_3 27025380

[8] Chen C Sun C Li J Ji X Wang Y Song C. Characterisation of *Staphylococcus aureus* isolates from bovine mastitis in Ningxia, Western China. J Glob Antimicrob Resist. (2021) 25:232–7. 10.1016/j.jgar.2021.03.021 33866044

[9] Yang F Zhang S Shang X Li H Zhang H Cui D. Short communication: Detection and molecular characterization of methicillin-resistant *S. aureus* isolated from subclinical bovine mastitis cases in China. J Dairy Sci. (2020) 103:840–5. 10.3168/jds.2019-16317 31733844

[10] Yang FQ Wang X Wang L Wang X Li J Luo S. Genetic characterization of antimicrobial resistance in *Staphylococcus aureus* isolated from bovine mastitis cases in north-west China. Integr. Agric. (2016) 15:2842–7. 10.1016/S2095-3119(16)61368-0

[11] Lerch MF Schoenfelder SMK Marincola G Wencker FDR Eckart M Förstner KU. non–coding RNA from the intercellular adhesion (ica) locus of staphylococcus epidermidis controls polysaccharide intercellular adhesion (PIA)-mediated biofilm formation. Mol Microbiol. (2019) 111:1571–91. 10.1111/mmi.14238 30873665

[12] Mirzaee M Najar–Peerayeh S Behmanesh M Moghadam MF. Relationship between adhesin genes and biofilm formation in vancomycin-intermediate *Staphylococcus aureus* clinical isolates. Curr Microbiol. (2015) 70:665–70. 10.1007/s00284-014-0771-9 25572495

[13] Kerro Dego O van Dijk JE Nederbragt H. Factors involved in the early pathogenesis of bovine *Staphylococcus aureus* mastitis with emphasis on bacterial adhesion and invasion. A review Vet Q. (2002) 24:181–98. 10.1080/01652176.2002.9695135 12540135

[14] Castilho IG Dantas STA Langoni H Araújo JP Jr Fernandes A Jr Alvarenga FCL. Host–pathogen interactions in bovine mammary epithelial cells and HeLa cells by *Staphylococcus aureus* isolated from subclinical bovine mastitis. J Dairy Sci. (2017) 100:6414–21. 10.3168/jds.2017-12700 28571985

[15] Zaatout N Ayachi A Kecha M. Interaction of primary mammary bovine epithelial cells with biofilm-forming staphylococci associated with subclinical bovine mastitis. Iran J Vet Res. (2019) 20:27–32. 10.22099/ijvr.2019.5139 31191696 PMC6509907

[16] Valle J Latasa C Gil C Toledo–Arana A Solano C Penadés JR Lasa I. Bap, a biofilm matrix protein of Staphylococcus aureus prevents cellular internalization through binding to GP96 host receptor. PLoS Pathog. (2012) 8:e1002843. 10.1371/journal.ppat.1002843 22876182 PMC3410863

[17] Oliveira M Bexiga R Nunes SF Vilela CL. Invasive potential of biofilm-forming Staphylococci bovine subclinical mastitis isolates. J Vet Sci. (2011) 12:95–7. 10.4142/jvs.2011.12.1.95 21368569 PMC3053474

[18] Wang X Teng D Wang X Hao Y Chen H Mao R. Internalization, distribution, and activity of peptide H2 against the intracellular multidrug-resistant bovine mastitis-causing bacterium S. aureus. Sci Rep. (2019) 9:7968. 10.1038/s41598-019-44459-x 31138863 PMC6538662

[19] Wellnitz O Bruckmaier RM. The innate immune response of the bovine mammary gland to bacterial infection. Vet J. (2012) 192:148–52. 10.1016/j.tvjl.2011.09.013 22498784

[20] Zhao X Lacasse P. Mammary tissue damage during bovine mastitis: causes and control. J Anim Sci. (2008). 86(13. Suppl):57–65. 10.2527/jas.2007-0302 17785603

[21] Zhang W Li X Xu T Ma M Zhang Y Gao MQ. Inflammatory responses of stromal fibroblasts to inflammatory epithelial cells are involved in the pathogenesis of bovine mastitis. Exp Cell Res. (2016) 349:45–52. 10.1016/j.yexcr.2016.09.016 27680776

[22] Bannerman DD Paape MJ Lee JW Zhao X Hope JC Rainard P. Escherichia coli and *Staphylococcus aureus* elicit differential innate immune responses following intramammary infection. Clin Diagn Lab Immunol. (2004) 11:463–72. 10.1128/CDLI.11.3.463-472.2004 15138171 PMC404560

[23] Ma M Pei Y Wang X Feng J Zhang Y Gao MQ. LncRNA XIST mediates bovine mammary epithelial cell inflammatory response via NF–κB/NLRP3 inflammasome pathway. Cell Prolif. (2019) 52:e12525. 10.1111/cpr.12525 30362186 PMC6430464

[24] Wesson CA Deringer J Liou LE Bayles KW Bohach GA Trumble WR. Apoptosis induced by *Staphylococcus aureus* in epithelial cells utilizes a mechanism involving caspases 8 and 3. Infect Immun. (2000) 68:2998–3001. 10.1128/IAI.68.5.2998-3001.2000 10769002 PMC97517

[25] Hu Q Cui X Tao L Xiu L Wang T Wang X. *Staphylococcus aureus* induces apoptosis in primary bovine mammary epithelial cells through Fas–FADD death receptor–linked caspase-8 signaling. DNA Cell Biol. (2014) 33:388–97. 10.1089/dna.2013.2195 24564258

[26] Chen Y Jing H Chen M Liang W Yang J Deng G. Transcriptional profiling of exosomes derived from *S. aureus*-infected bovine mammary epithelial cell line MAC–T by RNA-Seq Analysis. Oxid Med Cell Longev. (2021) 2021:8460355. 10.1155/2021/8460355 34367468 PMC8342165

[27] Jin W Ibeagha–Awemu EM Liang G BeauDOIn F Zhao X Guan le L. Transcriptome microRNA profiling of bovine mammary epithelial cells challenged with Escherichia coli or *S aureus* bacteria reveals pathogen directed microRNA expression profiles. BMC Genomics. (2014) 15:181. 10.1186/1471-2164-15-181 24606609 PMC4029070

[28] Sacco SC Velázquez NS Renna MS Beccaria C Baravalle C Pereyra EAL. Capacity of two *S. aureus* strains with different adaptation genotypes to persist and induce damage in bovine mammary epithelial cells and to activate macrophages. Microb Pathog. (2020) 142:104017. 10.1016/j.micpath.2020.104017 32006636

[29] Parkhomchuk D Borodina T Amstislavskiy V Banaru M Hallen L Krobitsch S. Transcriptome analysis by strand-specific sequencing of complementary DNA. Nucleic Acids Res. (2009) 37:e123. 10.1093/nar/gkp596 19620212 PMC2764448

[30] Goldstein LD Cao Y Pau G Lawrence M Wu TD Seshagiri S. Prediction and quantification of splice events from RNA-Seq Data. PLoS ONE. (2016) 11:e0156132. 10.1371/journal.pone.0156132 27218464 PMC4878813

[31] Hu C Zhang X Wei W Zhang N Wu H Ma Z. Matrine attenuates oxidative stress and cardiomyocyte apoptosis in doxorubicin-induced cardiotoxicity via maintaining AMPKα/UCP2 pathway. Acta Pharm Sin B. (2019) 9:690–701. 10.1016/j.apsb.2019.03.003 31384530 PMC6664099

[32] Genestier AL Michallet MC Prévost G Bellot G Chalabreysse L Peyrol S. *Staphylococcus aureus* panton-valentine leukocidin directly targets mitochondria and induces bax-independent apoptosis of human neutrophils. J Clin Invest. (2005) 115:3117–27. 10.1172/JCI22684 16276417 PMC1265849

[33] Löffler B Tuchscherr L Niemann S Peters G. *Staphylococcus aureus* persistence in non-professional phagocytes. Int J Med Microbiol. (2014) 304:170–6. 10.1016/j.ijmm.2013.11.011 24365645

[34] Moormeier DE Bayles KW. *Staphylococcus aureus* biofilm: a complex developmental organism. Mol Microbiol. (2017) 104:365–76. 10.1111/mmi.13634 28142193 PMC5397344

[35] Tuchscherr L Löffler B Buzzola FR Sordelli DO. *Staphylococcus aureus* adaptation to the host and persistence: role of loss of capsular polysaccharide expression. Future Microbiol. (2010) 5:1823–32. 10.2217/fmb.10.147 21155664

[36] Atalla H Gyles C Mallard B. Persistence of a *S.* aureus small colony variants (*S. aureus* SCV) within bovine mammary epithelial cells. Vet Microbiol. (2010) 143:319–28. 10.1016/j.vetmic.2009.11.030 20022186

[37] Jin D Ojcius DM Sun D Dong H Luo Y Mao Y. Leptospira interrogans induces apoptosis in macrophages via caspase-8- and caspase-3-dependent pathways. Infect Immun. (2009) 77:799–809. 10.1128/IAI.00914-08 19029301 PMC2632035

[38] Cai M Fan W Li X Sun H Dai L Lei D. The regulation of *Staphylococcus aureus*-induced inflammatory responses in bovine mammary epithelial cells. Front Vet Sci. (2021) 8:683886. 10.3389/fvets.2021.683886 34136558 PMC8200483

[39] Lim KH Staudt LM. Toll-like receptor signaling. Cold Spring Harb Perspect Biol. (2013) 5:a011247. 10.1101/cshperspect.a011247 23284045 PMC3579400

[40] Newton K Dixit VM. Signaling in innate immunity and inflammation. Cold Spring Harb Perspect Biol. (2012) 4:a006049. 10.1101/cshperspect.a006049 22296764 PMC3282411

[41] Luo J Manning BD Cantley LC. Targeting the PI3K-Akt pathway in human cancer: rationale and promise. Cancer Cell. (2003) 4:257–62. 10.1016/S1535-6108(03)00248-4 14585353

[42] Ruland J. Return to homeostasis: downregulation of NF-κB re-sponses. Nat Immunol. (2011) 12:709–14. 10.1038/ni.2055 21772279

[43] Lai JL Liu YH Liu C Qi MP Liu RN Zhu XF. Indirubin inhibits LPS–induced inflammation via TLR4 abrogation mediated by the NF-kB and MAPK signaling pathways. Inflammation. (2017) 40:1–12. 10.1007/s10753-016-0447-7 27718095

[44] Boaru SG Borkham–Kamphorst E Van de Leur E Lehnen E Liedtke C Weiskirchen R. NLRP3 inflammasome expression is driven by NF-κB in cultured hepatocytes. Biochem Biophys Res Commun. (2015) 458:700–6. 10.1016/j.bbrc.2015.02.029 25686493

[45] Zeng L Zou W Wang G. Cellular prion protein (PrP(C)) and its role in stress responses. Int J Clin Exp Med. (2015) 8:8042–50. PMID: 26221369; PMCID: PMC4509314. 26221369 PMC4509314

[46] Makrinou E Collinge J Antoniou M. Genomic characterization of the human prion protein (PrP) gene locus. Mamm Genome. (2002) 13:696–703. 10.1007/s00335-002-3043-0 12514748

[47] Díaz–San Segundo F Salguero FJ de Avila A Espinosa JC Torres JM Brun A. Distribution of the cellular prion protein (PrPC) in brains of livestock and domesticated species. Acta Neuropathol. (2006) 112:587–95. 10.1007/s00401-006-0133-1 16957924

[48] Wang C Wu R Li FD Liu L Zhang XL Zhao CL. Expression patterns of prion protein gene in differential genotypes sheep: quantification using molecular beacon real-time RT–PCR. Virus Genes. (2011) 42:457–62. 10.1007/s11262-011-0579-7 21318242

[49] Mehrpour M Codogno P. Prion protein: from physiology to cancer biology. Cancer Lett. (2010) 290:1–23. 10.1016/j.canlet.2009.07.009 19674833

[50] Linden R Martins VR Prado MA Cammarota M Izquierdo I Brentani RR. Physiology of the prion protein. Physiol Rev. (2008) 88:673–728. 10.1152/physrev.00007.2007 18391177

[51] Chiesa R Harris DA. Fishing for prion protein function. PLoS Biol. (2009) 7:e75. 10.1371/journal.pbio.1000075 19338390 PMC2661967

[52] Konturek PC Bazela K Kukharskyy V Bauer M Hahn EG Schuppan D. Helicobacter pylori upregulates prion protein expression in gastric mucosa: a possible link to prion disease. World J Gastroenterol. (2005) 11:7651–6. 10.3748/wjg.v11.i48.7651 16437693 PMC4727223

[53] Ingram RJ Isaacs JD Kaur G. A role of cellular prion protein in programming T-cell cytokine responses in disease. FASEB J. (2009) 23:1672–84. 10.1096/fj.08-116087 19204074

[54] Watarai M Kim S Erdenebaatar J Makino S Horiuchi M Shirahata T. Cellular prion protein promotes brucella infection into macrophages. J ExpMed. (2003) 198:5–17. 10.1084/jem.20021980 12847134 PMC2196088

[55] Ding T Zhou X Kouadir M Shi F Yang Y Liu J. Cellular prion protein participates in the regulation of inflammatory response and apoptosis in BV2 microglia during infection with *Mycobacterium bovis*. J Mol Neurosci. (2013) 51:118–26. 10.1007/s12031-013-9962-2 23345082

